# Slc38a9 Deficiency Induces Apoptosis and Metabolic Dysregulation and Leads to Premature Death in Zebrafish

**DOI:** 10.3390/ijms23084200

**Published:** 2022-04-11

**Authors:** Xiya Wu, Jianyang Chen, Chengdong Liu, Xuan Wang, Huihui Zhou, Kangsen Mai, Gen He

**Affiliations:** 1 Key Laboratory of Mariculture, Ministry of Education, Ocean University of China, Qingdao 266003, China; 17806278390@163.com (X.W.); chenjianyang@genomics.cn (J.C.); xuanwang@ouc.edu.cn (X.W.); zhouhuihui@ouc.edu.cn (H.Z.); kmai@ouc.edu.cn (K.M.); hegen@ouc.edu.cn (G.H.); 2 Key Laboratory of Aquaculture Nutrition and Feeds, Ministry of Agriculture, Ocean University of China, Qingdao 266003, China; 3 Laboratory for Marine Fisheries Science and Food Production Processes, Qingdao National Laboratory for Marine Science and Technology, Qingdao 266003, China

**Keywords:** SLC38A9, apoptosis, amino acid homeostasis, glycolysis, hypoxia

## Abstract

Eukaryotic cells control nutritional homeostasis and determine cell metabolic fate through a series of nutrient transporters and metabolic regulation pathways. Lysosomal localized amino acid transporter member 9 of the solute carrier family 38 (SLC38A9) regulates essential amino acids’ efflux from lysosomes in an arginine-regulated fashion. To better understand the physiological role of SLC38A9, we first described the spatiotemporal expression pattern of the *slc38a9* gene in zebrafish. A quarter of *slc38a9*^−/−^ mutant embryos developed pericardial edema and died prematurely, while the remaining mutants were viable and grew normally. By profiling the transcriptome of the abnormally developed embryos using RNA-seq, we identified increased apoptosis, dysregulated amino acid metabolism, and glycolysis/gluconeogenesis disorders that occurred in *slc38a9*^−/−^ mutant fish. *slc38a9* deficiency increased whole-body free amino acid and lactate levels but reduced glucose and pyruvate levels. The change of glycolysis-related metabolites in viable *slc38a9*^−/−^ mutant fish was ameliorated. Moreover, loss of *slc38a9* resulted in a significant reduction in hypoxia-inducible gene expression and hypoxia-inducible factor 1-alpha (Hif1α) protein levels. These results improved our understanding of the physiological functions of SLC38A9 and revealed its indispensable role in embryonic development, metabolic regulation, and stress adaption.

## 1. Introduction

The availability and quality of nutrients play a fundamental role in embryo development. Nutrients provide energy for cellular activity through catabolism and supply constituents for cellular biomass synthesis. Severe malnutrition may cause early embryonic mortality or affect human postnatal health [1]. Nutrient homeostasis is regulated by multiple mechanisms and signaling pathways [2]. For example, the mechanistic target of rapamycin complex 1 (mTORC1) dominates general protein synthesis through the phosphorylation of p70S6 kinase (S6K) and eIF4E binding protein (4EBP1). It integrates nutrient availability and growth factors to control cellular metabolism and growth. The full activation of mTORC1 requires its lysosomal localization. Amino acids, building blocks for protein synthesis, are one of the essential macronutrients for embryos’ development. The presence of nutrients, especially amino acids, induces mTORC1 to translocate to lysosomal membranes for activation [3]. How amino acids are sensed remained elusive until a series of nutrient sensors was recently discovered. Sestrin2, previously found as a stress-induced protein, binds intracellular leucine to transduce nutrient availability information [4,5]. Secretion associated Ras related GTPase 1B (SAR1B) is another novel leucine sensor but with a much higher affinity to leucine [6]. Cytosolic arginine sensor for mTORC1 subunit 1 (CASTOR1) is an intracellular arginine sensor that regulates mTORC1 signaling through a mechanism similar to sestrin2 and SAR1B [7]. In contrast to the above cytosolic amino acid sensors, SLC38A9, however, responds to lysosomal arginine levels specifically [8,9].

SLC38A9 was first identified as a potential amino acid transporter belonging to the slc38 family. It has a broad expression profile but a relatively low expression in rat tissues when compared with other slc38 proteins [10]. In contrast to SLC38A1-5, which localizes to the plasma membrane [11], and SLC38A10, which localizes to ER and Golgi [12], SLC38A9, as well as SLC38A7, are lysosomal membrane proteins [8,13]. SLC38A9 is a highly glycosylated transmembrane protein and consists of eleven transmembrane helices with a 120-residue N-terminal region towards the cytoplasm. Amino acid transport assay using proteoliposomes indicated that SLC38A9 is a bidirectional amino acid transporter [8]. SLC38A9 and Ragulator function together as noncanonical guanine exchange factors (GEF) to promote the activation of Rag GTPase heterodimers [14] and regulate mTORC1 signaling. In addition, SLC38A9 interacts with cholesterol through its conserved cholesterol-responsive motifs and mediates cholesterol-induced mTORC1 activation, independent of arginine sensing [15]. SLC38A9 also contributes to the sensing of lysine by the mTORC1 pathway [8].

The property of being able to sense a wide range of nutrients implies a potential role for SLC38A9 in maintaining cellular homeostasis. SLC38A9 was downregulated in drug-resistant ovarian cancer cell lines [16] and was a potential clinical target for mTORC1 deregulation-associated disease [8]. A recent study found that SLC38A9 also mediated endomembrane damage-promoted autophagy to maintain endomembrane homeostasis through the GATOR system [17]. As a newly discovered protein that orchestrates nutrient availability, cytoplasmic endomembrane homeostasis, and mTORC1 activity, the in vivo physiological role of SLC38A9 is rarely reported. To explore the intrinsic function of SLC38A9 during embryonic development, we took advantage of zebrafish embryos, which are feasible to image and manipulate due to their external development and transparency. The constant steady supply of nutrients from yolk cells makes the study of gene function free from external nutritional fluctuations. Furthermore, the crystal structure of zebrafish SLC38A9 is already solved [18]. In this study, we revealed the expression profile of *slc38a9* and generated *slc38a9*^−/−^ mutant zebrafish using CRISPR/Cas9. *slc38a9* deficiency caused pericardial edema and premature death. Transcriptional profiling revealed that SLC38A9 plays a critical role in amino acid and glucose metabolism. In addition, loss of *slc38a9* resulted in a significant reduction in hypoxia-inducible gene expression and Hif1α protein levels. It improved our understanding of the stress response function of SLC38A9.

## 2. Results

### 2.1. Spatiotemporal Expression Pattern of slc38a9 in Zebrafish

**Figure 1:**
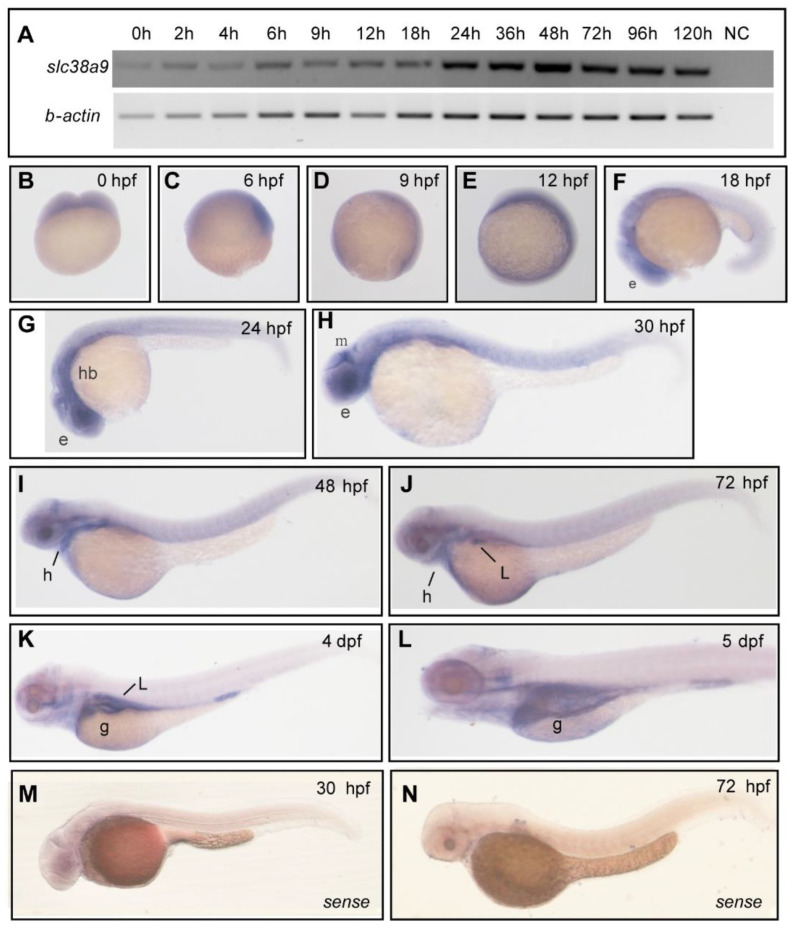
Spatiotemporal expression pattern of *slc38a9* in zebrafish. (**A**) RT-PCR analysis of zebrafish *slc38a9* mRNA at the indicated embryonic stages. Numbers indicate different developmental stages as hours post-fertilization. NC, negative control. *β-actin* as the internal control. (**B**–**L**) Wholemount in situ hybridization (WISH) reveals the *slc38a9* mRNA expression pattern in the zebrafish embryos at the indicated embryonic stages. *slc38a9* expression is ubiquitous from the one-cell stage to 12 hpf (**B**–**E**), restricted to the retina at 18 (**F**), 24 (**G**), and 30 hpf (**H**). The *slc38a9* expression encompasses the heart and midbrain region at 30 (**H**) and 48 hpf (**I**). From 72 hpf to 5 dpf, the slc38a9 expression is restricted to the liver and gut (**J**,**L**). (**M**,**N**) Views of zebrafish embryos hybridized with sense zebrafish *slc38a9* probe. Abbreviations: e, eye, mb, midbrain; g, gut; hb, hindbrain; l, liver; h, heart.

To evaluate the function of SLC38A9 in early development, we first examined the spatiotemporal expression pattern of *slc38a9* using qRT-PCR and whole-mount in situ hybridization. *slc38a9* mRNA was detectable in the first 4 h post-fertilization (hpf), indicating that *slc38a9* is a maternal mRNA. Zygotically expressed *slc38a9* reached its peak at 24 hpf and remained highly expressed after that (Figure 1A). Spatially, the expression of *slc38a9* was diffused across the whole embryo before 18 hpf. Interestingly, we found *slc38a9* mRNA was then highly expressed in tissues including the optic primordium, midbrain, hindbrain, and heart at 48 hpf (Figure 1B–H). Specific expression in the heart, optic primordium, brain, and liver were observed at 48–72 hpf (Figure 1I,J). Strong expression in the gut was also detected at 4–5 dpf (Figure 1L,M).

### 2.2. Knockout slc38a9 Causes Developmental Defects and Premature Death in Zebrafish

**Figure 2:**
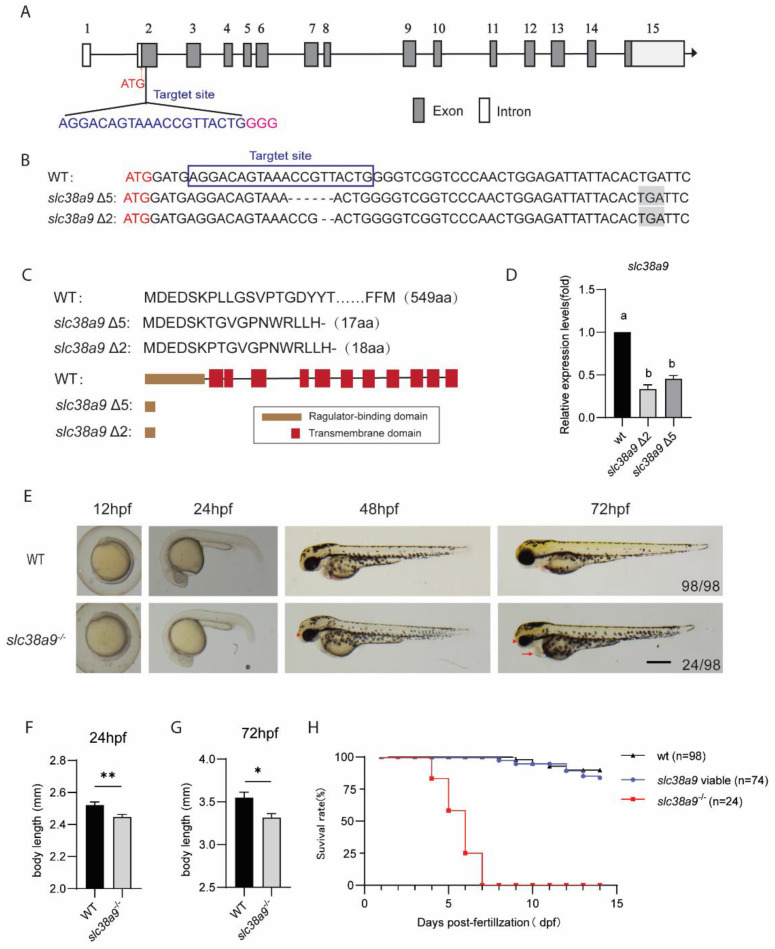
Knockout *slc38a9* cause developmental defects in zebrafish. (**A**) Zebrafish *slc38a9* gene structure. The selected Cas9 target sites sequence (blue) is in the second exon of the zebrafish *slc38a9* gene. The PAM sequence is colored in pink. (**B**) The sequencing trace data of wild type and *slc38a9* mutant alleles. The black shade is a premature stop codon. (**C**) The upper panel is the SLC38a9 protein sequences of WT and mutants. The lower panel is the schematic of the predicted SLC38A9 protein domains of WT and mutants. (**D**) Relative expression levels of *slc38a9* mRNA in mutant and WT zebrafish at 3 dpf. The mRNA levels of *slc38a9* were measured by qRT-PCR and normalized by *β-actin* mRNA levels. Data shown are mean ± SEM, *n* = 3. Different letters indicate significant differences at *p* < 0.05. (**E**) Live images of WT and mutant at the indicated developmental stage. The Arrowhead indicates the smaller eyes of the mutant, and arrow indicates the edematous pericardial cavity of the mutant. Scale bar = 500 μm. (**F**, **G**) The body length of mutant zebrafish was shorter than WT at 24 and 72 hpf. Data shown are mean ± SEM, *n* = 10. * *p* < 0.05, ** *p* < 0.01. (**H**) Viability of wild type and *slc38a9* mutant larvae.

The specific expression pattern of *slc38a9* implies that it may play an indispensable role during development. We then generated the *slc38a9* mutant using CRISPR/Cas9 (Figure 2A). Two independent *slc38a9*^−/−^ alleles were obtained. The *slc38a9* Δ5 and Δ2 alleles harbor five and two base pair (bp) deletions, respectively (Figure 2B). Both mutants introduce premature stop codons at the N-terminal domain and lack all transmembrane domains (Figure 2C). The qRT-PCR assay indicated clear RNA decay (Figure 2D). No morphological difference was observed at 12 and 24 hpf between wide type and *slc38a9* Δ5 alleles. However, 24 out of 98 mutant zebrafish exhibited pericardial edema and small eyes at 48 hpf; the abnormal phenotypes became severe at 72 hpf (Figure 2E). Similar results were also observed in the *slc38a9* Δ2 allele (Figure S1). The *slc38a9* Δ5 line was referred to as *slc38a9*^−/−^ mutant fish hereafter, and was used in subsequent studies unless otherwise stated. The overall body length of *slc38a9*^−/−^ mutant was decreased at 24 and 72 hpf (Figure 2F,G). While *slc38a9*^−/−^ mutant larvae with development defects died at 4–7 dpf, those normally developed *slc38a9*^−/−^ mutants (defined as *slc38a9*^−/−^ viable) have the same viability as wide type and exhibit normal fertility (Figure 2H).

### 2.3. Transcriptional Profiling of slc38a9^−/−^ Mutant

**Figure 3:**
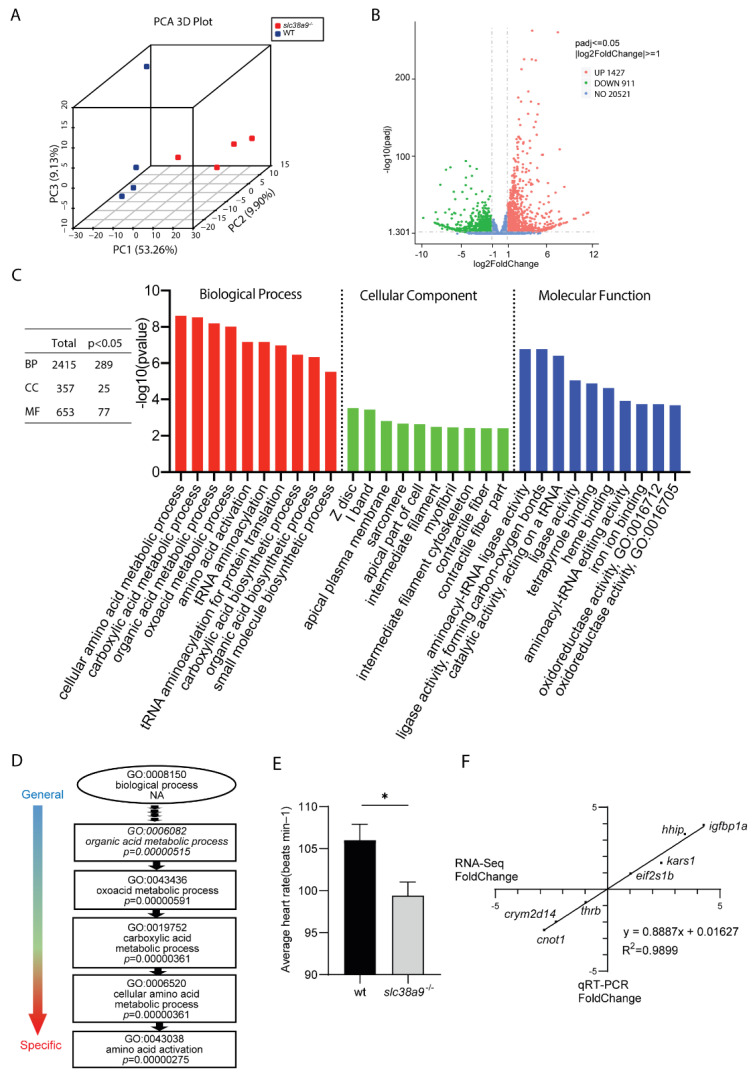
RNA sequencing analysis of *slc38a9* mutant zebrafish. (**A**) Principal component analysis (PCA) plot of four wild type and *slc38a9* mutant RNA-seq datasets. Principal component 1 (PC1), principal component 2 (PC2), and principal component 3 (PC3) were used for analysis. (**B**) Volcano plot of differential expression analysis of *slc38a9* mutant and control larvae showing the relationship between −log10(padj) and log2FoldChanges. The red dot shows upregulated genes, the green dot shows downregulated genes, and the blue dot shows an unchanged gene. (**C**) Gene ontology (GO) analysis of differentially expressed genes in biological processes (red column), cellular component (green column), and molecular function (blue column). (**D**) The directed acyclic graph (DAG) of biological process. The lower terms are more specific, while the upper terms are more general. (**E**) Average heart rate of 60 hpf WT and *slc38a9* mutant zebrafish larvae. Data shown are mean ± SEM, n = 10. * *p* < 0.05. (**F**) Correlation between qRT-PCR and RNA-seq results for select DEGs. The fold change values derived from the RNA-seq analysis of DEGs are compared with those obtained by qRT-PCR determined by 2^−ΔΔCT^. The reference line indicates the expected linear relationship.

To identify affected networks that cause developmental defects in *slc38a9*^−/−^ mutants, we particularly sampled *slc38a9*^−/−^ mutants with abnormal phenotypes at 60 hpf for high-throughput RNA-seq. A number of 41.14–47.91 million (M) raw reads of each sample were collected (Table S1). An amount of 92.72–93.49% clean reads were mapped to the zebrafish genome (GRCz11) using HISAT2 with a unique map between 78.21 and 79.1%. Finally, 22, 859 protein codon genes were identified and used in the following analysis. Principal component analysis (PCA) showed that the first (PC1) and second (PC2) principal components explained 53.26 and 9.9% of the variance, respectively (Figure 3A). By comparing two genotypes, a total of 2338 differentially expressed genes (DEG) were identified with 1427 being upregulated and 911 being downregulated in *slc38a9* mutants (Figure 3B). The DEGs were assigned to biological process (BP), cellular component (CC), and molecular function (MF) gene ontology (GO) categories. Totals of 289 BP, 25 CC, and 77 MF terms were significantly affected (Figure 3C, left panel). The top 10 affected GO terms are shown in Figure 3C. DEGs annotated as GO terms related to “cellular amino acid metabolic process”, “carboxylic acid metabolic process”, “organic acid metabolic process”, “oxoacid metabolic process”, and “amino acid activation” were the most significantly enriched in biological processes (Figure 3C). We found “amino acid activation” to be the most specific biological process GO term by DAG analysis (Figure 3D). DEGs annotated as GO terms related to “Z disc”, “I band”, “apical plasma membrane”, “sarcomere”, and “apical part of cell” were the most significantly enriched in the biological process (Figure 3C). These GO terms were highly related to muscle contraction. We then asked if the cardiac rhythm was affected. The results showed that knockout of slc38a9 significantly reduced heart rate (Figure 3E). DEGs annotated as GO terms related to “aminoacyl-tRNA ligase activity”, “ligase activity, forming carbon-oxygen bonds”, “catalytic activity, acting on a tRNA”, “ligase activity”, and “tetrapyrrole binding” were the most significantly enriched in biological process (Figure 3C). The linear regression analysis between RNA-seq and qRT-PCR was performed to validate the RNA-seq data (r = 0.9899, *p* < 0.0001) (Figure 3F).

### 2.4. Slc38a9 Deficiency Induces Apoptosis and Alters Multiple Metabolic Pathways

**Figure 4:**
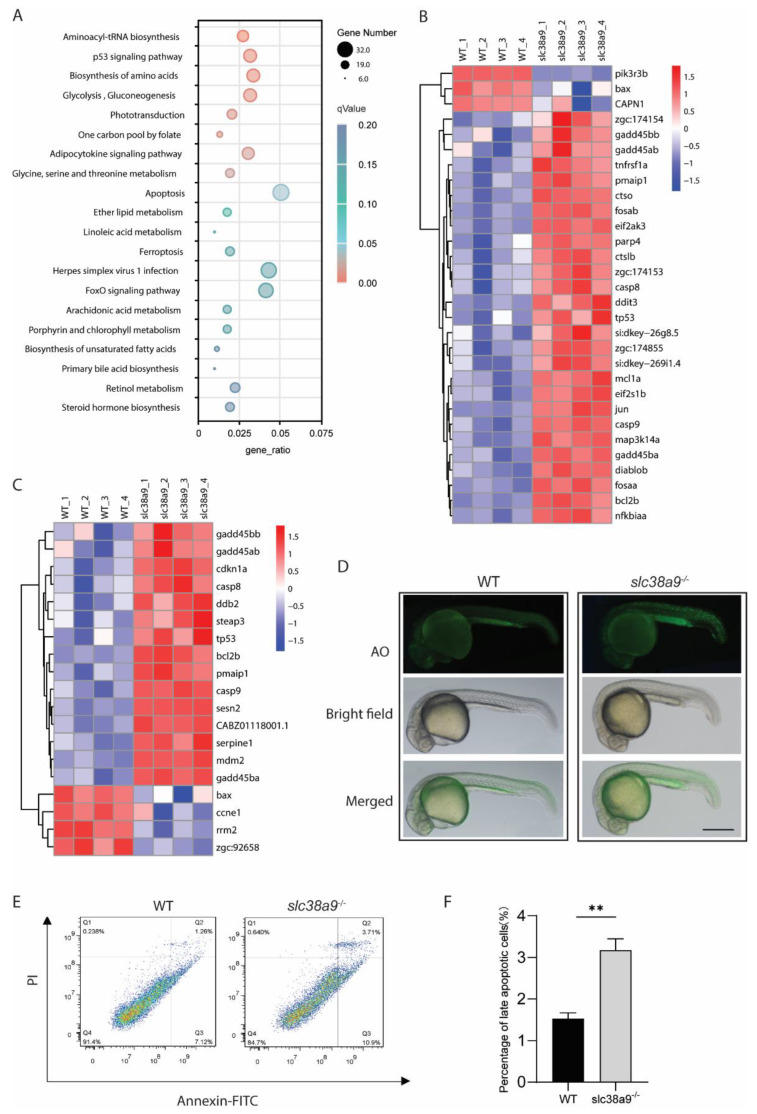
*Slc38a9* Deficiency induces apoptosis and alters multiple metabolic pathways. (**A**) Kyoto Encyclopedia of Genes and Genomes (KEGG) enrichment analysis of differentially expressed genes. The X-axis indicates gene ratio, the size of dots represents the number of genes in the pathway, and the color indicates the size of the qValue. (**B**) Heatmaps of transcripts in apoptosis enrichment. (**C**) Heatmaps of transcripts in p53 signaling pathway enrichment. (**D**) Larvae were stained with AO to visualize apoptotic cells identified as green punctate dots. Scale bar = 500μm. (**E**) Annexin/PI apoptosis analysis in WT and *slc38a9* mutant zebrafish embryos (3 dpf). (**F**) The percentage of late apoptosis cells in WT and *slc38a9* mutant zebrafish embryos. Data shown are mean ± SEM, *n* = 3. ** *p* < 0.01.

To further reveal the mechanisms by which *slc38a9* deficiency leads to embryonic lethality, the annotated DEGs were applied to Kyoto Encyclopedia of Genes and Genomes (KEGG) analysis. The results showed that although 149 KEGG pathways were enriched using DEGs, only 9 KEGG pathways were significantly affected when using padj <0.05 as a threshold (Table S2). These KEGG pathways were categorized into five classes, including “Genetic Information Processing” (dre00970: Aminoacyl-tRNA biosynthesis), “Cellular Processes” (dre04115: p53 signaling pathway, dre04210: Apoptosis), “Metabolism” (dre01230: Biosynthesis of amino acids, dre00010: Glycolysis / Gluconeogenesis, dre00670: One carbon pool by folate, dre00260: Glycine, serine, and threonine metabolism), “Organismal Systems” (dre04744: Phototransduction), and “Organismal Systems” (dre04920: Adipocytokine signaling pathway) (Figure 4A). In the apoptosis-related DEGs, both caspase8 (*casp8*) and caspase9 (*casp9*) were upregulated (Figure 4B). Unexpectedly, BCL-2 family gene *bcl2b* was upregulated, whereas *bax* was downregulated. The expression of *pmaip1* (orthologous to human PMAIP1/NOXA), another pro-apoptotic BCL-2 family gene, was upregulated. The elevated levels of *eif2ak3*, *eif2s1b*, and *ddit3* indicated that ER stress occurred. Death receptor *tnfrsf1a* and its downstream effectors were also upregulated, such as *jun* and *fosaa*. In addition, a decrease in the pro-survival gene *pik3r3b* was observed (Figure 3B). The expression of multiple p53 target genes and p53 itself was enhanced, whereas the cell cycle regulator *ccne1*, a regulatory subunit of CDK2, was downregulated (Figure 3C). AO staining results showed more apoptosis cells in *slc38a9*^−/−^ mutants (Figure 3D). Apoptosis was further confirmed and quantified by a flow cytometer. The proportion of apoptosis cells was increased from 1.53 to 3.18% in *slc38a9*^−/−^ mutants (Figure 3E,F).

### 2.5. Biosynthesis of Amino Acids and Aminoacyl-tRNA Were Enhanced in slc38a9^−/−^ Embryos

**Figure 5:**
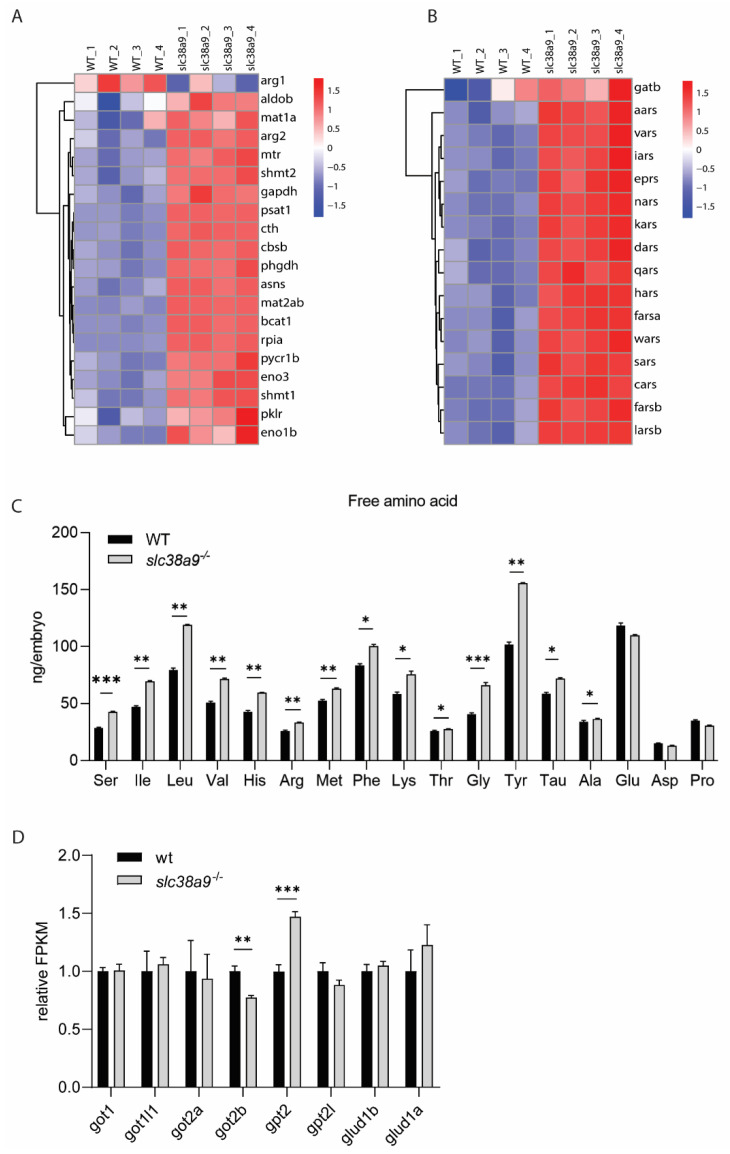
Biosynthesis of amino acids and aminoacyl-tRNA were enhanced in *slc38a9*^−/−^ embryos. (**A**) Heatmaps of transcripts in the biosynthesis of amino acids’ enrichment. (**B**) Heatmaps of transcripts in aminoacyl-tRNA synthetases’ enrichment. (**C**) Most free amino acids (FAAs) increased in *slc38a9*^−/−^ mutants. The contents of most FAAs (Leu, Met, Ile, Val, Lys, etc.) increased except for Pro, Glu, and Asp. The profile of FAAs was measured using the automated amino acid analyzer. (**D**) The relative FPKM of major aminotransferases in WT and *slc38a9* mutant zebrafish embryos (60 hpf). Data shown are mean ± SEM, *n* = 3. * *p* < 0.05, ** *p* < 0.01, *** *p* < 0.005.

SLC38A9 was originally identified as an amino acid transporter; its deficiency may lead to dysregulation of amino acid metabolism. KEGG analysis revealed that amino acid biosynthesis pathways were significantly affected in *slc38a9*^−/−^ mutant (Figure 5A). Furthermore, enhanced aminoacyl-tRNA synthetases were also observed (Figure 5B). Branched-chain amino-acid transaminase (*bcat1*), which facilitates the reversible transamination of BCAAs, was upregulated. Accordingly, Ile, Leu, and Val cellular levels were significantly increased (Figure 5C). Both *mtr* (5-methyltetrahydrofolate-homocysteine methyltransferase) and its product Met were upregulated. However, the expression of methionine adenosyltransferase *mat1a* and *mat2ab* were also increased, indicating a high turnover rate of Met. Serine hydroxymethyltransferase *shmt1* and *shmt2*, which regulate the serine–glycine conversion, were upregulated. Cth catalyzes the last step in the transsulfuration pathway from methionine to cysteine, and Asns catalyzes the synthesis of the non-essential amino acid Asn from aspartate. Asp and Gln are two critical downstream targets of amino acid response (AAR) signaling pathway. We found the expression of both *cth* and *asns* was enhanced. Arginase-1 (Arg1) and Arginase-2 (Arg2) convert Arg to urea and ornithine. Our result showed while *arg1* is downregulated, *arg2* and free Arg levels were increased. Among the 17 free amino acids detected, 14 amino acids were upregulated in *slc38a9*^−/−^ mutants, whereas the level of Glu, Asp, and Pro were identical to wild-type. Since the total free amino acids level were modified, we then asked if amino acid catabolism were affected by investigating the expression of major aminotransferases and glutamate dehydrogenases. As shown in Figure 5D, glutamic–oxaloacetic transaminase 2b (*got2b*) was downregulated, whereas alanine aminotransferase 2 (*gpt2*) was upregulated. These results indicate that knockout of *slc38a9* leads to amino acid metabolism disorders.

### 2.6. Slc38a9 Regulates Glycolysis and Gluconeogenesis in Zebrafish Embryo

**Figure 6:**
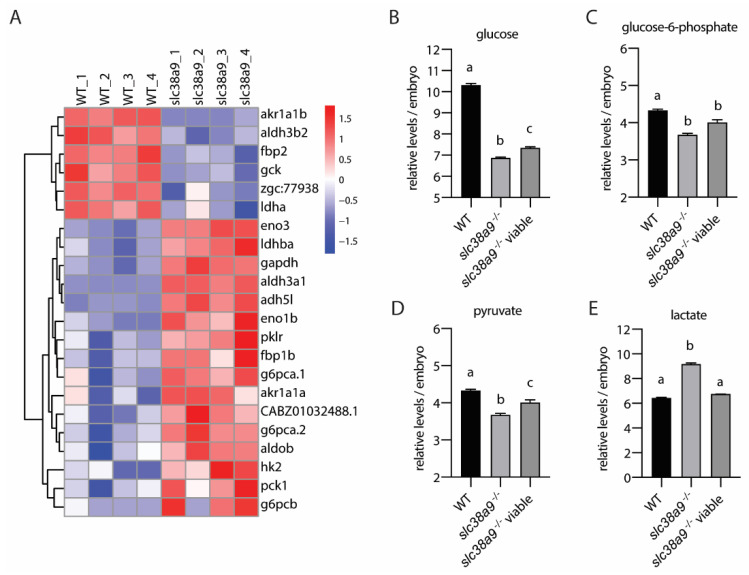
*Slc38a9* regulates glycolysis and gluconeogenesis in zebrafish embryos. (**A**) Heatmaps of transcripts in glycolysis and gluconeogenesis enrichment. (**B**–**E**) Concentrations of glucose (**B**), glucose-6-phosphate (**C**), pyruvate (**D**), and lactate (**E**) in WT and *slc38a9* mutant zebrafish embryos (60 hpf) measured by LC-MS/MS. Data shown are mean ± SEM, *n* = 3. Different letters indicate significant differences at *p* < 0.05.

Twenty-two DEGs related to glycolysis and gluconeogenesis metabolism pathways, with six DEGs being downregulated and sixteen DEGS being upregulated (Figure 6A). The hexokinases *gck* and *hk2* were oppositely regulated in *slc38a9*^−/−^ mutants (Figure 6A). Whole-body glucose levels, detected using LC/MS in *slc38a9* mutants with severe phenotype, was dramatically lower than in wild type; however, the viable *slc38a9*^−/−^ mutant had a higher glucose level than abnormal *slc38a9*^−/−^ mutants. Similar results were also observed in glucose-6-phosphate levels. Fructose-bisphosphate aldolase B (*aldob*), glyceraldehyde-3-phosphate dehydrogenase (*gapdh*), enolase 1a (*eno1a*), and enolase 3 (*eno3*) are enzymes that can catalyze both glycolysis and gluconeogenesis. Their expression in *slc38a9*^−/−^ mutants was significantly increased (Figure 6A). Glucose-6-phosphatase catalytic subunits (*g6pca.1*, *g6pca.2*, *g6pcb*), fructose-1,6-bisphosphatase (*fbp1b*, *fbp2*), and phosphoenolpyruvate carboxykinase (*pck1*) are rate-limiting enzymes that are involved in gluconeogenesis. Most of these genes were upregulated except for *fbp2*, which was downregulated (Figure 6A). Pyruvate kinase (*pklr*) is the enzyme involved in the last step of glycolysis to produce pyruvate, and its expression was upregulated. Nevertheless, pyruvate content was reduced in *slc38a9*^−/−^ mutants. However, this reduction was ameliorated in viable *slc38a9*^−/−^ mutants (Figure 6D). In addition, the level of lactate, the final product of anaerobic glycolysis, was significantly elevated in abnormal *slc38a9*^−/−^ mutants but not in viable *slc38a9*^−/−^ mutants (Figure 6E).

### 2.7. Slc38a9 Deficiency Leads to Dysregulated Hypoxia Response

**Figure 7:**
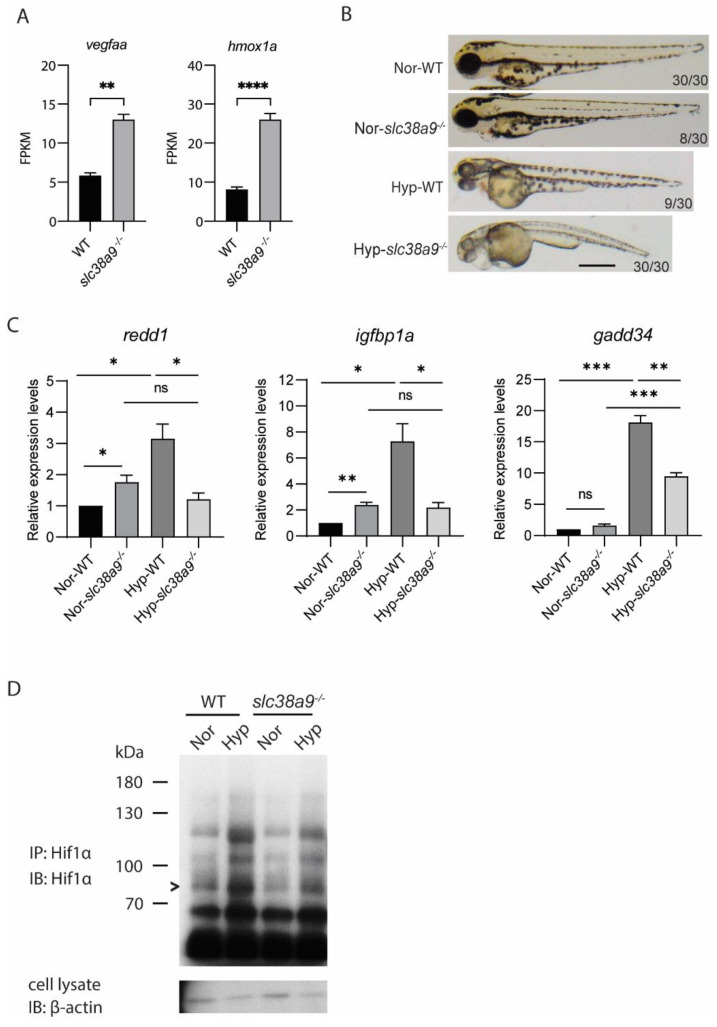
*Slc38a9* deficiency leads to dysregulated hypoxia response. (**A**) The FPKM of hypoxia-induced genes in WT and *slc38a9* mutant zebrafish embryos (60 hpf). (**B**) The phenotypic penetrance of the *slc38a9* mutant increased under hypoxia treatment. Zebrafish larvae (24 hpf) were treated with hypoxia embryo rearing solution (DO = 1 mg/L) for 10 h. Normoxia (Nor) and hypoxia (Hyp). (**C**) The hypoxia-induced increase of Hif1α target genes was blocked in the *slc38a9* mutant zebrafish. The experimental groups were the same as described in (**B**). The expression of Hif1α target genes was detected by qRT-qPCR and normalized by the *β-actin* mRNA levels. The mRNA levels of Hif1α target genes were upregulated in WT zebrafish larvae under hypoxic conditions, while this increase was completely blocked in the *slc38a9* mutant. Data shown are mean ± SEM, n = 3. * *p* < 0.05, ** *p* < 0.01, *** *p* < 0.005, **** *p* < 0.001. (**D**) The increase of Hif1α protein level was attenuated in *slc38a9* mutants under hypoxic conditions. The experimental groups were the same as described in (**B**). The immunoprecipitation was used to enrich the Hif1α protein. The representative result is shown in (**D**). > Hif1α.

Increased lactate is common during stress conditions [19]. RNA-seq results showed a significant increase in the expression of *vegfaa* and *hmox1a*, two stress-induced genes (Figure 7A). We then asked if *slc38a9*^−/−^ mutant is less tolerant to environmental stresses such as hypoxia, which is a typical stress often faced by aquatic animals. Treating embryos with hypoxic conditions could further increase the phenotypic penetrance of *slc38a9*^−/−^ mutation (Figure 7B). Organisms respond to oxygen deficiency by initiating the expression of a series of genes through the key transcription factors Hif (hypoxia-inducible factors). By detecting the expression of Hif target genes, we found both *redd1* and *igfbp1a* transcriptional levels were elevated under normoxic conditions in *slc38a9*^−/−^ mutant. While hypoxia significantly increased *redd1* and *igfbp1a* expression in wild type, this hypoxia-induced increase was completely blocked by *slc38a9*^−/−^ knockout (Figure 7C). The expression of *gadd34* was also reduced, although to a lesser extent (Figure 7C). We then measured the protein levels of Hif1α under different conditions. By immunoprecipitation, we were able to enrich and detect Hif1α protein. We found that the basal protein level of Hif1α in *slc38a9*^−/−^ mutant is lower than in wild type. While hypoxia increased Hif1α protein level in wild type embryos, this increase was attenuated in *slc38a9*^−/−^ mutants (Figure 7D). These results indicated that loss of *slc38a9* inhibited the response to hypoxia.

## 3. Discussion

The specific subcellular localization and structural characteristics make SLC38A9 a unique lysosomal arginine sensor. Accumulated evidence indicates that SLC38A9 plays important roles in amino acid efflux from lysosomes, mTORC1 activation, and tumor growth using cultured cells and the orthotopic allograft model [8,9,20,21]. To further reveal the physiological function of SLC38A9, the *slc38a9* mutant zebrafish was generated in the present study. We found *slc38a9*^−/−^ mutant embryos presented with small eye size, pericardial edema, and metabolic disorder. The pericardial edema became much more severe at 4–7 dpf. Moreover, swim bladder inflation was disrupted at 5 dpf in *slc38a9*^−/−^ mutants (data not shown), indicating that the swimming activity may be reduced. Knockdown of *lat3*, a Na^+^-independent neutral l-amino acid transporter, also resulted in pericardial edema by 96 hpf in zebrafish [22]. Pericardial edema was generally associated with heart failure [23,24]. We indeed found reduced heartbeat rate in *slc38a9*^−/−^ mutants, and GO enrichment indicated affected heme binding and iron ion binding, hinting that SLC38A9 might be involved in normal heart development. However, pericardial edema was considered as a non-specific phenotype due to its high incidence in genetic mutants and pharmacological studies [23]. The known function of SLC38A9 is regulating mTOR signaling and transporting amino acids. The I68A variant of SLC38A9 mutants cannot bind Rag–Ragulator to control the mTOR signaling pathway but can transport arginine similarly to wild-type SLC38A9 [8], whereas the T133W mutant loses the ability to interact and transport amino acids [21]. Studying both mutants in *slc38a9*-null fish needs to be done to further reveal the distinct function of SLC38A9 in vivo.

KEGG analysis revealed that the expression of multiple genes which are involved in apoptosis and p53 signaling pathways were affected in *slc38a9*^−/−^ mutants. Paradoxically, the anti-apoptotic BCL-2 family gene *bcl2b* was upregulated, whereas the pro-apoptotic gene *bax* was downregulated. One possible explanation is that stresses induced by gene mutation imposed selective pressure for elevated levels of pro-survival proteins, such as BCL-2. Cells with elevated BCL-2 can be more susceptible to apoptosis than normal cells [25]. The lack of Sesn2, an intracellular leucine sensor, in mice also results in apoptosis in dendritic cells, although Sesn2-null mice are fully viable [4]. Apoptosis is readily induced by inhibition of mTOR [26], which is intrinsically downstream of amino acid sensors, including SLC38A9. Unexpectedly, the mTORC1 signaling was not enriched by KEGG analysis. mTOR is essential for embryonic development. mTOR-null is lethal in mice, and their development is arrested at E5.5 [27]. Whether lack of *slc38a9* in zebrafish causes dysregulation of mTOR signaling need to be further tested.

A study in HEK293 cells found that loss of SLC38A9 had no effect on the whole-cell amino acid level but enormously boosted the lysosomal concentrations of several amino acids, including most non-polar, essential amino acids [21]. Using an in vivo model, we were able to quantify the effect of SLC38A9 on whole-body free amino acid homeostasis. According to our results, while most amino acid levels were increased, Glu and Asp levels weren’t changed in the *slc38a9* mutant. This might be due to the reduced expression of got, which catalyzes the convention of Glu and Asp, and enhanced expression of *gpt*, which facilitates the Glu to Ala conversion. Fish uptake and degrade yolk proteins or free amino acids to meet their amino acid requirements during development [28]. Autophagy plays an essential role in yolk nutrition absorption in zebrafish [29]. Although overexpression of SLC38A9 inhibits autophagy in HEK293T, the SLC38A9-null cells did not have a defect in autophagy activation [9,21]. *slc38a9* deficiency elevating free amino acid levels in zebrafish may also be due to reducing protein synthesis caused by impaired mTOR activation [9,21]. Thus, whether the elevated whole-body free amino acid levels in *slc38a9* mutant fish are caused by increased absorption and/or reduced utilization need to be further determined.

Another finding in the present study is only a quarter of the offspring of *slc38a9* mutants showed abnormal phenotype. The penetrance of the mutant is modulated by inherited factors and, in some cases, environmental factors [30]. Reduced penetrance was found in human hereditary diseases and zebrafish mutants [31,32]. Loss of *igfbp5a*, for example, induces premature death of zebrafish, whereas this mutant doesn’t show overt phenotypes when raised in calcium-rich solutions [32]. We found acute hypoxia treatment increased the proportion of abnormal phenotypes to 100% and further aggravated the abnormal phenotype, implying that SLC38A9 plays a critical role in acute hypoxia tolerance. Interestingly, the expression of redd1 and igfbp1a were increased under normoxic culture conditions. Given the fact that different stresses can induce both genes’ expression, we proposed that the *slc38a9*^−/−^ mutant was facing chronic stress. Redd1 can be induced by DNA damage, excess ROS, and ER stress [32]. In addition, Redd1 can be induced by energetic stresses, including glucose deprivation [33]. Our data indeed showed that the glucose levels in *slc38a9*^−/−^ mutant decreased, which might be due to elevated glycolysis.

The expression of glycolysis and gluconeogenesis-related genes are differently regulated in *slc38a9*^−/−^ mutants. Interestingly, some genes belonging to the same protein family showed opposite expression patterns. For example, while the hexokinase gene *hk2* mRNA level was increased, the glucokinase (*gck*) mRNA level was reduced. Different hexokinases are expressed in a tissue-specific manner, and only HK2 is overexpressed in cancer cells, which might contribute to the high glycolytic rate in tumors [34]. We also found the expression of *fbp1b* was increased, and that of *fbp2* decreased. Mammalian FBP1 and FBP2 encode the liver and muscle isoforms of FBPase, respectively, and FBP1 is mainly expressed in gluconeogenic organs [35]. One primary source for gluconeogenesis is glycogenic amino acids. Whether elevated free amino acid levels increased in *slc38a9*^−/−^ mutant-induced gluconeogenesis is unclear.

The metabolite assay found dramatic differences between viable and lethal *slc38a9*^−/−^ mutants. The metabolite pattern of the viable mutant was more similar to that of the wild type, indicating that targeting metabolism may rescue the lethal phenotype of *slc38a9*^−/−^ mutants. In drosophila, methionine supply rescued the fecundity defect in aci-reductone dioxygenase 1 mutants [36]. Dietary supplementation of unsaturated fatty acids (UFAs) could rescue the lethal phenotype of desat1 mutants, a gene involved in drosophila UFA metabolism [37]. These results indicate that targeting metabolism is an efficient way to ameliorate phenotype caused by a metabolism gene mutant.

In summary, we revealed the spatiotemporal expression pattern of *slc38a9* in zebrafish and generated *slc38a9*^−/−^ mutant fish. We found that *slc38a9* deficiency results in severe pericardial edema and premature death. By performing whole-organism RNA-seq of wild type and *slc38a9*^−/−^ mutant fish, we identified increased apoptosis, dysregulated amino acid metabolism, and glycolysis/gluconeogenesis disorder in *slc38a9*^−/−^ mutant fish. We experimentally confirmed that free amino acids and lactate were accumulated in zebrafish. Moreover, elevated expression of stress-related genes and attenuated hypoxia tolerance were observed in *slc38a9*^−/−^ mutant fish. The results of this study have revealed the indispensable function of *slc38a9* on embryonic development. Nevertheless, whether the phenotype observed in *slc38a9*^−/−^ mutant fish was caused by the direct or indirect effects of *slc38a9* deficiency is unclear. Future studies are needed to determine the mechanism by which SLC38A9 regulates embryonic development.

## 4. Materials and Methods

### 4.1. Experimental Animals

Zebrafish (*Danio rerio*) were maintained in a circulation water system on a 14:10 h darkness cycle at 28 °C and fed twice daily. Fertilized eggs were obtained by natural breeding. They were raised at 28.5 °C in an embryo rearing solution (ERS, 5 mmol/L NaCl, 0.33 mmol/L MgSO_4_, 0.33 mmol/L CaCl_2_, 0.17 mmol/L KCl, pH 7.2) and staged according to the standard method [38]. All experiments were conducted following the guidelines approved by the Animal Care Committee of Ocean University of China (Permit Number: 11001).

### 4.2. slc38a9 Knockout by CRISPR/Cas9 System

The zebrafish *slc38a9* mutants were generated by the CRISPR/Cas 9 system. The dual NLS-tagged Cas9 mRNA was synthesized in vitro using the linearized pSP6-2sNLS-spCas9 as templates with SP6/T7 mMESSAGE mMACHINE Kit (Ambion, Carlsbad, CA, USA) according to the manufacturer’s instruction. For making *slc38a9* sgRNA (5′-AGGACAGTAAACCGTTACTG-3′), the transcription template DNA was amplified from the plasmid pT7-gRNA, a plasmid containing the T7 promoter, gRNA sequence, and the tracrRNA sequences. The primers used for PCR amplified are listed in Table S3. To obtain injected F0 embryos, we collected the wild-type embryos and microinjected them with 1nl of Cas9 mRNA (500 ng/μL) and *slc38a9* sgRNA (100 ng/μL) at one-cell stage embryos. PCR was performed for genotyping using the primers in Table S3, and the products were analyzed by DNA sequencing. Finally, two independent *slc38a9* mutant lines were obtained. Most of the assays were performed with the zebrafish of the *slc38a9*Δ5 unless specifically indicated.

### 4.3. Hypoxia Treatment

We pumped N_2_ gas into ERS until the dissolved oxygen (DO) = 1 mg/L to generate hypoxic ERS (DO = 1 mg/L). The dissolved oxygen of ERS was detected by the DO sensor (Xylem, Munich, Germany). Then, we transferred fifteen 24 hpf embryos and 2 mL hypoxic ERS into a 35 mm culture dish, which was placed into a modular hypoxic chamber (MIC-101, Billups-Rothenberg, San Diego, CA, USA). The chamber was sealed and placed into a thermo-static incubator at 28.5 °C.

### 4.4. Quantitative RT-PCR (qRT-PCR)

Total RNA was isolated from fifteen embryos using TRIzol reagent and reverse-transcribed to cDNA using M-MLV reverse transcriptase (Takara, Shiga, Japan). qRT-PCR was carried out using TB Green Premix Taq (Takara, Shiga, Japan) with a real-time PCR detection system (Bio-Rad, Hercules, CA, USA). All primer sequences of target genes are listed in the Table S3. The expression level of a particular gene transcript was calculated based on the standard curve and normalized by the *β-actin* levels, as no expression changes of *β-actin* were observed in zebrafish among different treatments. The gene expression levels were calculated by a 2^−ΔΔCT^ method. The data were reported as fold increases of the control.

### 4.5. Whole-Mount In Situ Hybridization

In situ hybridization was performed as previously reported [39]. Briefly, *slc38a9* mRNA was detected using a digoxigenin (DIG)-labeled antisense riboprobe. After incubation in a byan alkaline phosphatase-conjugated anti-DIG antibody (Roche, Switzerland), larvae were stained in nitroblue tetrazolium (NBT) /5-Bromo-4-chloro-3-indolyl phosphate (BCIP) (Roche, Basel, Switzerland) substrate. After staining, they were washed in 1 × PBST. Images were acquired using Nikon stereomicroscopes (Nikon Inc., Melville, NY, USA).

### 4.6. High-Throughput RNA Sequencing

Total RNA was isolated from pools of 10 larvae (60 hpf) by standard extraction methods. The quality of each sample was determined by an Agilent2100 bioanalyzer (Agilent Technologies Inc., Santa Clara, CA, USA) and agarose gel electrophoresis. The RNA quality score (RIN/RQN) was 10 for all of the samples.

The mRNA with polyA tail was enriched by Oligo (dT) magnetic beads, and cDNA libraries were constructed using the NEBNext^®^ Ultra™ RNA Library Prep Kit for Illumina^®^ (NEB, Beverly, MA, USA). The sequencing of these cDNA libraries was performed on the Novogene HiSeq4000 sequencing platform (Novogene, Beijing, China) according to the manufacturer’s stand protocol.

### 4.7. Bioinformatics Analysis of RNA Sequence Data

Raw reads were tested for quality control and filtered into clean reads. The clean reads were aligned with the zebrafish genome *Danio rerio*, GRCz11) using HISAT (v2.0.4) to obtain the localization information of reads on the reference genome. The gene expression was quantified using the FPKM method (fragment per kilobase of transcript per million fragments sequenced) [40]. Differentially expressed genes were identified by DESeq2 software [41]. Genes with fold change >2 and adjusted *p*-value ≤ 0.05 are statistically significant. The GO (gene ontology) functional enrichment analysis and KEGG (Kyoto Encyclopedia of Genes and Genomes) pathway enrichment analysis were performed using clusterProfile software. The threshold of significant enrichment was padj < 0.05.

### 4.8. AO Staining

Zebrafish were incubated in a culture medium containing 2 µg/mL acridine orange (AO) (Sigma-Aldrich, St. Louis, MO, USA) for 30 min. Before observation, embryos were washed in fresh ERS three times and anesthetized by 0.08% MS-222. Images were captured using a microscope equipped with a Nikon DS-U3 camera (Nikon Inc., Melville, NY, USA). NIS-Elements software (Nikon Inc., Melville, NY, USA) was used.

### 4.9. Apoptosis Analysis by Annexin V-FITC/PI Staining

Quantitative analysis of apoptosis in the single cells of zebrafish embryos was done using the Annexin V-FITC/PI kit (Beyotime, Shanghai, China). Using recombinant Annexin V conjugated to green-fluorescent FITC dye, the kit detects the internalization of phosphatidyl-serine in the apoptotic cells, and dead cells are detected using propidium iodide (PI) that binds to exposed DNA. Briefly, zebrafish larvae (3 dpf, *n* = 30/group) were digested with Liberase TM (Roche, Basel, Switzerland) for 30 min at 28.5 °C into single-cell isolation. Then, the cell supernatant was filtered through 40 μm mesh and stained using FITC-Annexin V/PI and analyzed in a flow cytometer. All the experiments were performed in triplicates. Data were analyzed using FlowJo (v7.6) software (Tree Star Inc. Ashland, OR, USA).

### 4.10. Free Amino Acid Analysis

An L-8900 automated amino acid analyzer (Hitachi, Tokyo, Japan) analyzed zebrafish’s free amino acid concentration with a high-performance lithium column. Seventy embryos of 60 hpf for each group were homogenized in 200 μL 10% sulfosalicylic acid solution. After centrifugation at 13,000 rpm for 15 min, the supernatant was filtered through 0.22 μm filters for free amino acid measurement.

### 4.11. Mass Spectrometry

Sixty zebrafish embryos were transferred to a 2 mL quenching solution (40:40:20 Methanol:Acetonitrile:H_2_O, 0.1 M Formic acid, 15% NH_4_HCO_3_) homogenized at −20 °C. After centrifugation at 13,000 rpm for 15 min, the supernatants were analyzed using a QTRAP5500 LC-MS/MS mass spectrometer (AB Sciex, Framingham, MA, USA) in positive/negative mode and with multiple reaction monitoring (MRM) scan type. An amount of 5 μL of metabolite extracts were injected on an amide column (100 × 2.1 mm, 1.7 μm; Waters, Milford, MA, Ireland) using a mobile phase A (water, 5% acetonitrile, 20 mM ammonium acetate, 20 mM NH_4_OH, pH 9.4) and mobile phase B (100% acetonitrile) at a constant flow rate of 300 μL/min; initial conditions: 95%B, 1–12 min: B goes from 95% linearly to 55%, 12–13 min: B goes from 55% linearly to 40%, 13–15 min: 40%B, 15–15.1 min: B goes from 40% linearly to 95%, 15.1–18 min: 95%B. The raw data were processed and analyzed using SCIX OS (AB Sciex, Framingham, MA, USA) and Markerview1.3.1 software (AB Sciex, Framingham, MA, USA).

### 4.12. Western Blot and Immunoprecipitation

Sixty zebrafish embryos were sonicated in Precooled Lysis Buffer (50 mM Tris-HCl (pH 8.0), 150 mM NaCl, 1% NP-40, 1 mM EGTA, five tablets of phosphatase inhibitor, and two tablets of EDTA-free protease inhibitor (Roche, Basel, Switzerland) per 50 mL)). Lysates were cleared by centrifugation in a microcentrifuge (13,000 rpm, 10 min, 4 °C). Proteins were quantified with BCA (Beyotime, Shanghai, China). For immunoprecipitations, lysates were incubated with HIF1α primary antibody (#NB100-479, Novus Biologicals, Littleton, CO, USA) and protein A/G-sepharose (Santa Cruz Biotechnology, Inc., Santa Cruz, CA, USA) (overnight with rotation, 4 °C). Beads were recovered and washed four times with PBS before analysis by SDS–PAGE and immunoblotting.

### 4.13. Statistical Analysis

Values are presented as mean ± standard error of the mean (SEM). Statistical significance among experimental groups was determined using one-way ANOVA followed by Tukey’s multiple comparison test. Statistical tests were performed using GraphPad Prism 8 software (GraphPad Software, San Diego, CA, USA), and significance was defined as *p* < 0.05 or greater. Correlation analysis was carried out by fitting the data to the equation Y = X to calculate *r*^2^ using GraphPad Prism 8 (GraphPad Software, San Diego, CA, USA).

## Data Availability

The data presented in this study are available on request from the corresponding author.

## Supplementary Materials

The following are available online at https://www.mdpi.com/article/10.3390/ijms23084200/s1.

## References

[1] Velazquez M.A. (2015). Impact of maternal malnutrition during the periconceptional period on mammalian preimplantation embryo development. Domest. Anim. Endocrinol..

[2] Efeyan A., Comb W.C., Sabatini D.M. (2015). Nutrient-sensing mechanisms and pathways. Nature.

[3] Sancak Y., Peterson T.R., Shaul Y.D., Lindquist R.A., Thoreen C.C., Bar-Peled L., Sabatini D.M. (2008). The Rag GTPases bind raptor and mediate amino acid signaling to mTORC1. Science.

[4] Budanov A.V., Karin M. (2008). p53 target genes sestrin1 and sestrin2 connect genotoxic stress and mTOR signaling. Cell.

[5] Wolfson R.L., Chantranupong L., Saxton R.A., Shen K., Scaria S.M., Cantor J.R., Sabatini D.M. (2016). Sestrin2 is a leucine sensor for the mTORC1 pathway. Science.

[6] Chen J., Ou Y., Luo R., Wang J., Wang D., Guan J., Li Y., Xia P., Chen P.R., Liu Y. (2021). SAR1B senses leucine levels to regulate mTORC1 signalling. Nature.

[7] Chantranupong L., Scaria S.M., Saxton R.A., Gygi M.P., Shen K., Wyant G.A., Wang T., Harper J.W., Gygi S.P., Sabatini D.M. (2016). The CASTOR Proteins Are Arginine Sensors for the mTORC1 Pathway. Cell.

[8] Wang S., Tsun Z.-Y., Wolfson R.L., Shen K., Wyant G.A., Plovanich M.E., Yuan E.D., Jones T.D., Chantranupong L., Comb W. (2015). Lysosomal amino acid transporter SLC38A9 signals arginine sufficiency to mTORC1. Science.

[9] Rebsamen M., Pochini L., Stasyk T., de Araujo M.E., Galluccio M., Kandasamy R.K., Snijder B., Fauster A., Rudashevskaya E.L., Bruckner M. (2015). SLC38A9 is a component of the lysosomal amino acid sensing machinery that controls mTORC1. Nature.

[10] Sundberg B.E., Wååg E., Jacobsson J.A., Stephansson O., Rumaks J., Svirskis S., Alsiö J., Roman E., Ebendal T., Klusa V. (2008). The evolutionary history and tissue mapping of amino acid transporters belonging to solute carrier families SLC32, SLC36, and SLC38. J. Mol. Neurosci..

[11] Mackenzie B., Erickson J.D. (2004). Sodium-coupled neutral amino acid (System N/A) transporters of the SLC38 gene family. Pflug. Arch..

[12] Tripathi R., Hosseini K., Arapi V., Fredriksson R., Bagchi S. (2019). SLC38A10 (SNAT10) is Located in ER and Golgi Compartments and Has a Role in Regulating Nascent Protein Synthesis. Int. J. Mol. Sci..

[13] Chapel A., Kieffer-Jaquinod S., Sagné C., Verdon Q., Ivaldi C., Mellal M., Thirion J., Jadot M., Bruley C., Garin J. (2013). An extended proteome map of the lysosomal membrane reveals novel potential transporters. Mol. Cell Proteom..

[14] Shen K., Sabatini D.M. (2018). Ragulator and SLC38A9 activate the Rag GTPases through noncanonical GEF mechanisms. Proc. Natl. Acad. Sci. USA.

[15] Castellano B.M., Thelen A.M., Moldavski O., Feltes M., Van Der Welle R.E., Mydock-McGrane L., Jiang X., Van Eijkeren R.J., Davis O.B., Louie S.M. (2017). Lysosomal cholesterol activates mTORC1 via an SLC38A9–Niemann-Pick C1 signaling complex. Science.

[16] Januchowski R., Zawierucha P., Andrzejewska M., Ruciński M., Zabel M. (2013). Microarray-based detection and expression analysis of ABC and SLC transporters in drug-resistant ovarian cancer cell lines. Biomed. Pharm..

[17] Jia J., Abudu Y.P., Claude-Taupin A., Gu Y., Kumar S., Choi S.W., Peters R., Mudd M.H., Allers L., Salemi M. (2018). Galectins Control mTOR in Response to Endomembrane Damage. Mol. Cell.

[18] Lei H.T., Mu X., Hattne J., Gonen T. (2021). A conformational change in the N terminus of SLC38A9 signals mTORC1 activation. Structure.

[19] Garcia-Alvarez M., Marik P., Bellomo R. (2014). Stress hyperlactataemia: Present understanding and controversy. Lancet Diabetes Endocrinol.

[20] Lei H.-T., Ma J., Martinez S.S., Gonen T. (2018). Crystal structure of arginine-bound lysosomal transporter SLC38A9 in the cytosol-open state. Nat. Struct. Mol. Biol..

[21] Wyant G.A., Abu-Remaileh M., Wolfson R.L., Chen W.W., Freinkman E., Danai L.V., Vander Heiden M.G., Sabatini D.M. (2017). mTORC1 Activator SLC38A9 Is Required to Efflux Essential Amino Acids from Lysosomes and Use Protein as a Nutrient. Cell.

[22] Sekine Y., Nishibori Y., Akimoto Y., Kudo A., Ito N., Fukuhara D., Kurayama R., Higashihara E., Babu E., Kanai Y. (2009). Amino acid transporter LAT3 is required for podocyte development and function. J. Am. Soc. Nephrol..

[23] Narumanchi S., Wang H., Perttunen S., Tikkanen I., Lakkisto P., Paavola J. (2021). Zebrafish Heart Failure Models. Front Cell Dev. Biol..

[24] Zhu X.Y., Wu S.Q., Guo S.Y., Yang H., Xia B., Li P., Li C.Q. (2018). A Zebrafish Heart Failure Model for Assessing Therapeutic Agents. Zebrafish.

[25] Adams J.M., Cory S. (2018). The BCL-2 arbiters of apoptosis and their growing role as cancer targets. Cell Death Differ..

[26] He K., Zheng X., Li M., Zhang L., Yu J. (2016). mTOR inhibitors induce apoptosis in colon cancer cells via CHOP-dependent DR5 induction on 4E-BP1 dephosphorylation. Oncogene.

[27] Gangloff Y.G., Mueller M., Dann S.G., Svoboda P., Sticker M., Spetz J.F., Um S.H., Brown E.J., Cereghini S., Thomas G. (2004). Disruption of the mouse mTOR gene leads to early postimplantation lethality and prohibits embryonic stem cell development. Mol. Cell Biol..

[28] Yilmaz O., Patinote A., Com E., Pineau C., Bobe J. (2021). Knock out of specific maternal vitellogenins in zebrafish (Danio rerio) evokes vital changes in egg proteomic profiles that resemble the phenotype of poor quality eggs. BMC Genom..

[29] Moss J.J., Wirth M., Tooze S.A., Lane J.D., Hammond C.L. (2021). Autophagy coordinates chondrocyte development and early joint formation in zebrafish. FASEB J..

[30] Pei W., Williams P.H., Clark M.D., Stemple D.L., Feldman B. (2007). Environmental and genetic modifiers of squint penetrance during zebrafish embryogenesis. Dev. Biol..

[31] Zanon A., Pramstaller P.P., Hicks A.A., Pichler I. (2018). Environmental and Genetic Variables Influencing Mitochondrial Health and Parkinson’s Disease Penetrance. Parkinsons Dis..

[32] Liu C., Xin Y., Bai Y., Lewin G., He G., Mai K., Duan C. (2018). Ca(2+) concentration-dependent premature death of *igfbp5a*(−/−) fish reveals a critical role of IGF signaling in adaptive epithelial growth. Sci. Signal.

[33] Sofer A., Lei K., Johannessen C.M., Ellisen L.W. (2005). Regulation of mTOR and cell growth in response to energy stress by REDD1. Mol. Cell Biol..

[34] Wu J., Hu L., Wu F., Zou L., He T. (2017). Poor prognosis of hexokinase 2 overexpression in solid tumors of digestive system: A meta-analysis. Oncotarget.

[35] Liu G.M., Zhang Y.M. (2018). Targeting FBPase is an emerging novel approach for cancer therapy. Cancer Cell Int..

[36] Chou H.Y., Lin Y.H., Shiu G.L., Tang H.Y., Cheng M.L., Shiao M.S., Pai L.M. (2014). ADI1, a methionine salvage pathway enzyme, is required for Drosophila fecundity. J. Biomed. Sci..

[37] Bousquet F., Chauvel I., Flaven-Pouchon J., Farine J.P., Ferveur J.F. (2016). Dietary rescue of altered metabolism gene reveals unexpected Drosophila mating cues. J. Lipid. Res..

[38] Kimmel C.B., Ballard W.W., Kimmel S.R., Ullmann B., Schilling T.F. (1995). Stages of embryonic development of the zebrafish. Dev. Dyn..

[39] Liu C., Dai W., Bai Y., Chi C., Xin Y., He G., Mai K., Duan C. (2017). Development of a Whole Organism Platform for Phenotype-Based Analysis of IGF1R-PI3K-Akt-Tor Action. Sci. Rep..

[40] Trapnell C., Williams B.A., Pertea G., Mortazavi A., Kwan G., van Baren M.J., Salzberg S.L., Wold B.J., Pachter L. (2010). Transcript assembly and quantification by RNA-Seq reveals unannotated transcripts and isoform switching during cell differentiation. Nat. Biotechnol..

[41] Love M.I., Huber W., Anders S. (2014). Moderated estimation of fold change and dispersion for RNA-seq data with DESeq2. Genome Biol..

