# Supplementary material for: Slc38a9 Deficiency Induces Apoptosis and Metabolic Dysregulation and Leads to Premature Death in Zebrafish
Source: Int J Mol Sci. 2022 Apr 11;23(8):4200. doi: 10.3390/ijms23084200 (PMC9025135; doi:10.3390/ijms23084200)
Supplement: Supplementary file 1 [file ijms-23-04200-s001.zip › Supplemental Files/Supplemental Figure S1.pdf]

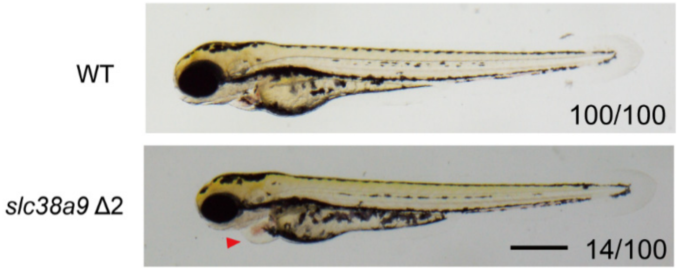

Figure S1. Live images of WT and slc38a9 Δ2 at 72 hpf. Arrowheads indicated the edematous pericardial cavity of mutants. Scale bar = 500 μm.
